# Honeybees fed D-galactose exhibit aging signs with changes in gut microbiota and metabolism

**DOI:** 10.1128/msystems.01487-24

**Published:** 2025-03-28

**Authors:** Guanzhou Zhou, Jiabin Hu, Mengqi Xu, Yiyuan Li, Ruqi Chang, Jiaqi Zeng, Wanyue Dan, Lihua Peng, Zikai Wang, Gang Sun, Fei Pan, Yunsheng Yang

**Affiliations:** 1 School of Medicine, Nankai University 481107 , Tianjin, Tianjin, China; 2 Microbiota Laboratory, Clinical Division of Microbiota, Department of Gastroenterology and Hepatology, The First Medical Center, Chinese PLA General Hospital 678390 , Beijing, Beijing, China; 3 Medical School of Chinese PLA 104607 , Beijing, Beijing, China; 4 National Clinical Research Center for Geriatric Diseases, Chinese PLA General Hospital 104607 , Beijing, Beijing, China; 5 State Key Laboratory of Kidney Diseases, The First Medical Center, Chinese PLA General Hospital 104607 , Beijing, Beijing, China; Chinese Academy of Sciences, Beijing, China

**Keywords:** *Apis mellifera*, D-galactose, aging, butyric acid, gut microbiota

## Abstract

Honeybees (*Apis mellifera*), as social insects, exhibit complex social behaviors and cognitive functions. The short lifespan and stable gut microorganisms of honeybees provide certain availability as a rapid and high-flux animal model for aging research. This study explored the effect of D-galactose, a common aging inducer, on honeybees and investigated the associated effects and mechanisms, with particular focus on the potential protective role of sodium butyrate. Experimental cohorts were established as follows: conventional (CV) group, D-galactose-treated (DG) group, and sodium butyrate-treated (SB) group. The CV group was fed sucrose solution; the DG group was fed D-galactose solution; and the SB group was fed D-galactose and sodium butyrate solution. A comprehensive assessment was conducted on day 15 post-treatment, including survival analysis, starvation test, motor, learning and memory ability tests, malondialdehyde test, and Smurf test. Potential mechanisms through the microbiome and metabolome were investigated. Compared to the honeybees from the CV group, those in the DG group showed a shortened lifespan, a weaker energy storage ability, impaired motor, learning, and memory abilities, reduced weight, increased oxidation, and a disrupted gut barrier. These phenotypic changes were associated with microbial dysbiosis characterized by *Lactobacillus* enrichment and diminished butyrate levels. Notably, sodium butyrate supplementation extended the honeybees’ lifespan and improved their learning and memory abilities damaged by D-galactose. Our findings establish honeybees as a valuable model system for aging research and highlight the crucial role of butyrate metabolism in senescence regulation.

**IMPORTANCE:**

This study presents a novel approach to investigating aging processes by establishing a D-galactose-induced aging model in honeybees. Our findings demonstrate that butyrate supplementation effectively attenuates D-galactose-induced senescence phenotypes, suggesting its potential as a therapeutic intervention for age-related decline. This research provides a unique model system for aging studies and highlights the significant role of butyrate in modulating senescence progression. The results contribute to our understanding of the molecular mechanisms underlying aging and offer new insights into potential anti-aging strategies.

## INTRODUCTION

Aging is a complex biological process characterized by the progressive deterioration of physiological functions, including diminished locomotor activity, cognitive decline, reduced stress resistance, and increased susceptibility to neurodegenerative disorders and other age-related pathologies (1). At the cellular and molecular levels, aging is marked by several hallmark features, including genomic instability, telomere attrition, epigenetic modifications, and loss of proteostasis. These alterations lead to dysregulated nutrient sensing, mitochondrial dysfunction, cellular senescence, and stem cell exhaustion, collectively contributing to the aging phenotype (2). The gastrointestinal tract serving as a primary interface for nutrient absorption, metabolic regulation, and immune homeostasis harbors a complex ecosystem of microorganisms and their metabolites, collectively referred to as the gut microbiome. This gut microbiome plays a crucial role in the aging process, with emerging evidence suggesting its involvement in modulating systemic inflammation, oxidative stress, immune responses, and metabolic homeostasis (3).

Honeybees (*Apis mellifera*) are widely recognized for their highly eusocial behavior, characterized by complex social organization, resource sharing, precise division of labor, and intricate communication. Compared with traditional small animal models, such as *Caenorhabditis elegans* and *Drosophila melanogaster*, honeybees offer distinct advantages for gut microbiota research, including the feasibility of generating germ-free individuals, a substantial intestinal capacity, and a highly diverse gut microbial community (4). Moreover, the completion of the honeybee genome project represents a milestone in honeybee research, enabling analyses of individual and colony characteristics at the genomic level (5). Notably, despite sharing identical genomes, queens and workers differ starkly in their reproductive capacity, behavior, and longevity (from 1 month for workers to 1 year for queens) primarily based on caste differentiation. This unique biological feature establishes honeybees as an exceptional model system for senescence (6, 7). The distinct biological attributes of queen bees offer valuable opportunities for investigating mechanisms underlying longevity, while the abbreviated lifespan of worker bees presents an advantageous model system for the high-throughput screening of pharmacological interventions targeting lifespan extension.

Since the seminal discovery that D-galactose administration accelerates aging phenotypes in murine models, chronic D-galactose treatment in rodents has been extensively utilized as an experimental model for aging research. This model reliably recapitulates the key aspects of human aging, including reduced lifespan, impaired motor and cognitive functions, neuronal degeneration, increased apoptosis, and dysregulation of gene expression patterns (8, 9). Building upon this foundation, we developed an innovative experimental paradigm to elucidate the role of gut microbiome in senescence progression. Specifically, we administered D-galactose to honeybees and conducted comprehensive analyses of gut microbiome alterations. Our findings not only reveal a pivotal role of butyrate in modulating the senescence process but also provide a novel framework for future aging research.

## MATERIALS AND METHODS

### Subjects and groups

Honeybees (*Apis mellifera*) were collected from brood frames in Pinggu District, Beijing, China, in May 2023. All bees used in this study came from the same colony. The generation of microbiota-free honeybees was performed following established protocols from our previous studies (10, 11). On day 0, germ-free honeybees were aseptically transferred to sterile rearing cages maintained at controlled environmental conditions (35°C, 50% relative humidity) and provisioned with sterile sucrose solution (50% [wt/vol]). The experimental cohort was systematically allocated into three treatment groups: (i) conventional honeybee group (control group, CV group), (ii) D-galactose treatment group (DG group), and (iii) sodium butyrate intervention group (SB group). Each treatment condition was replicated across multiple cup cages, with each cup containing approximately 25 honeybees.

On days 1 and 2, all experimental groups received a standardized diet consisting of 5 mL wild bee hindgut homogenate mixed with 700 µL 1× phosphate-buffered saline, 0.5 g sterilized pollen, and 700 µL sucrose solution (50%, wt/vol) to establish synchronized and normalized gut microbiota conditions. On days 3–9, dietary interventions were implemented as follows: the CV group received sterile pollen and sucrose solution (50%, wt/vol); the DG group was administered sterile pollen and D-galactose-supplemented sucrose solution (10% [wt/vol] D-galactose in 50% [wt/vol] sucrose solution); and the sodium butyrate (SB) group received sterile pollen and sodium butyrate-supplemented solution (50% [wt/vol] sucrose solution containing 10% [wt/vol] D-galactose and 10 mM sodium butyrate). On days 10–14, both the DG and SB groups were transitioned to the standard sucrose solution. All dietary preparations were administered through 2 mL tubes and changed every 24 h to ensure freshness. On day 15, comprehensive behavioral assessments were conducted across all experimental groups, followed by tissue collection of gut and brain samples for subsequent analytical procedures.

### Survival assays and starvation tests

Survival curves were constructed by systematically recording the initial population and daily mortality rates of bees within each cup cage at 24 h intervals. To evaluate the impact of nutritional stress, we conducted starvation resistance assays across all experimental groups. This assessment was based on the established principle that stress resistance capacity typically diminishes with aging in insects, leading to elevated mortality rates (12). Specifically, on day 15, honeybees were subjected to complete deprivation of sucrose solution and pollen while maintaining access to water to prevent dehydration. Mortality was monitored at 4 h intervals until complete cohort mortality was achieved. The experimental design incorporated 100 honeybees for both survival analysis and starvation resistance testing. Statistical comparisons of survival differences were performed using Kaplan–Meier survival analysis coupled with log-rank tests implemented in GraphPad Prism 9 (GraphPad Software, San Diego, CA, USA).

### Motor activity test

Motor activity tests were performed based on reports previously published by our team and Zaluski et al. (13, 14). The tests were performed in the laboratory using an opaque acrylic box (60 × 10 × 1.5 cm), which was divided into five lanes (50 × 1.7 × 1.5 cm). Individual bees were positioned at the starting point of each lane, confined by an opaque barrier. The terminal end of each lane was equipped with a fluorescent light source, serving as a phototactic stimulus, and covered with a transparent acrylic sheet to facilitate behavioral observation. Experiments were carried out under controlled dark conditions with the fluorescent illumination activated, thereby inducing positive phototactic responses in the bees. The time for each bee to traverse the 50 cm distance was systematically recorded. The experimental design included a cohort of 50 honeybees, with each individual undergoing five replicate trials to ensure data reliability.

### Learning and memory tests

Associative olfactory learning and memory tests based on proboscis extension response were performed in accordance with the methodology described in reports by our group (10, 11). The experiment utilized nonanol (conditioned stimulus [CS]; Sigma-Aldrich, St. Louis, MO, USA), sucrose solution (unconditioned stimulus [US]; 50%, wt/vol), and n-hexanal (novel odor [Nod]; Macklin, Shanghai, China) as odor sources. First, the honeybees were placed in a rearing box for 2 h of starvation. Then, individual honeybees were immobilized using custom-designed 0.8 mm wide-bullet shell harnesses secured with adhesive tape. At the beginning of each trial, the harnessed bee was placed inside the arena for 5 s to minimize contextual stress responses. The conditioning protocol involved presenting the nonanol odor (CS) to the antennae for 4 s, followed by a 3 s sucrose stimulation (US) with a 1 s temporal overlap. Sucrose delivery was precisely administered via syringe needle contact with the proboscis to elicit PER. The conditioning regimen consisted of five consecutive trials with 10 min inter-trial intervals to establish CS–US associations. Following the conditioning phase, all subjects underwent an additional 2 h starvation period before memory assessment. Memory retention was evaluated through a differential presentation of two olfactory stimuli: the conditioned stimulus (nonanol) and a novel odor (n-hexanal). Successful memory retention was operationally defined as exclusive proboscis extension to the conditioned nonanol stimulus. The experimental cohort comprised 60 honeybees that completed all testing procedures.

### Malondialdehyde test

Lipid peroxidation, serving as a biomarker of oxidative stress, was quantitatively assessed through the measurement of malondialdehyde (MDA) concentrations using the commercially available MDA Assay Kit (BC0025; Solarbio, Beijing, China) (15). Tissue samples, including head, thorax, and abdominal segments, were collected from 50 bees per experimental group and processed in accordance with the manufacturer’s protocol. The absorbance was measured in 96-well plates with the Cytation 5 Detector (Agilent, Santa Clara, CA, USA) at 532 nm. The presentation of the concentration of MDA was based on the liquid volume, which is proportional to the mass of samples.

### Smurf test

In accordance with our previously established methodology (10), a non-invasive "Smurf" assay was employed to evaluate intestinal barrier integrity, utilizing a cohort of 60 honeybees. The experimental procedure involved the administration of a non-toxic blue dye solution (1% brilliant blue in 50% sucrose solution) as a permeability marker. Phenotypic classification was based on dye distribution patterns: individuals exhibiting blue hemolymph or complete abdominal coloration were categorized as Smurf-positive (Smurf+), indicative of compromised intestinal permeability. Conversely, subjects displaying either a distinct blue gut outline or no coloration were classified as Smurf-negative (Smurf−), suggesting maintenance of intestinal barrier function. Quantitative assessment of Smurf-positive individuals within each cup cage was conducted on day 7 after the blue dye administration.

### Tissue collection

The intestine of honeybees, including the midgut, ileum, and rectum, was collected in a 1.5 mL sterile centrifuge tube, frozen in liquid nitrogen, and stored at −80°C. Brain samples were extracted under a dissecting microscope. After fixing on a beeswax plate using insect needles, the head cuticle of the bee was removed. The whole brain was placed on a glass slide and immersed in RNAlater solution (Thermo Fisher Scientific, Waltham, MA, USA). After removal of the glands and compound eyes, the isolated brain was flash-frozen in liquid nitrogen and stored at −80°C for subsequent analyses.

### Gut hematoxylin–eosin staining

The dissected intestine was fixed in 4% (wt/vol) paraformaldehyde overnight. After fixation, the intestine was dehydrated in a graded series of ethanol and xylene, embedded in paraffin, and then dried at 60°C. The paraffin-embedded tissue was cooled to complete solidification and sectioned at 4–6 mm thickness. After dewaxing and dehydration through a series of xylene and ethanol, the sections were stained with hematoxylin for 5 min and processed in 1% acidic ethanol (1% hydrochloric acid in 70% ethanol). The tissues were then rinsed in purified water, stained with eosin for 3 min, and rehydrated in an ethanol series and xylene. Slides were mounted with synthetic resin and examined under a microscope.

### Analysis of gene expression by RT-qPCR

Total RNA was isolated from individual brain and gut tissues using the Quick-RNA MiniPrep Kit (RN001; ES Science, Shanghai, China), followed by cDNA synthesis through reverse transcription with the Quick-RNA MiniPrep Kit (RT001; ES Science). Quantitative PCR was conducted on the ABI PlusOne Real-time System (StepOnePlus; Applied Biosystems, Inc., Foster City, CA, USA) utilizing SYBR Green enzyme (QP002; ES Science). Gene-specific primers, as detailed in the supplementary materials, were employed for target amplification. The 18S ribosomal RNA served as an endogenous control for normalization, and relative gene expression levels were determined using the 2^−ΔΔCt^ method. All samples were processed in parallel under identical amplification conditions, with each experimental group comprising five biological replicates.

### Gut DNA extraction and 16S rRNA sequencing

A sterile and disposable pestle was used to grind the entire digestive tract of the honeybee, and genomic DNA was extracted from each sample individually using the FastPure Stool DNA Isolation Kit (MJYH, Shanghai, China). Full-length 16S rRNA sequencing was performed at Majorbio Bio-Pharm Technology Co., Ltd. (Shanghai, China). The bacterial 16S rRNA genes were amplified using the universal bacterial primers 27F (5′-AGRGTTYGATYMTGGCTCAG-3′) and 1492R (5′-RGYTACCTTGTTACGACTT-3′). Purified products were pooled at equimolar concentrations, and a DNA library was constructed using the SMRTbell Prep Kit 3.0 (Pacific Biosciences, CA, USA). Purified SMRTbell libraries were sequenced on the PacBio Sequel IIe System (Pacific Biosciences). Ten biological replicates of CV and DG groups were processed for the analysis.

### Metabolomic analysis of the gut

Ten biological replicates from the CV and DG groups were conducted for untargeted metabolomic analysis. The liquid chromatography–mass spectrometry (LC–MS) analyses were performed using a 1290 Infinity II System (Agilent) with 6545 UHD and Accurate-mass Q-TOF mass spectrometers (Agilent) in positive and negative ionization modes by APExBIO Co., Ltd. (Shanghai, China).

### Measurement of butyric acid concentration

To determine the precise concentration of butyric acid in the CV and DG groups, targeted metabolomic analysis was performed on 10 biological replicates. Quality control samples consisting of predetermined concentrations of mixed standard solutions were utilized to evaluate the stability of the analytical system. Butyric acid was extracted from the samples and quantified using LC–MS/MS. The analysis was conducted on an ExionLC AD System interfaced with a QTRAP 6500+ mass spectrometer (Sciex, USA) at Majorbio Bio-Pharm Technology Co., Ltd. (Shanghai, China)

### Statistical analysis

All experimental data obtained from the honeybee studies were statistically processed and analyzed using GraphPad Prism 9 (GraphPad Software, San Diego, CA, USA). Parametric data characterized by normal distribution and homogeneity of variance were presented as mean ± standard deviation (SD) and subjected to independent samples *t*-test for comparative analysis. Non-parametric data sets were expressed as median values with interquartile ranges (IQR) and analyzed using the Mann–Whitney *U* test. Survival analysis and starvation resistance data were evaluated through Kaplan–Meier survival curves with log-rank test comparisons. The threshold for statistical significance was established at *P* < 0.05 for all analyses.

## RESULTS

### D-galactose shortened the lifespan of honeybees and weakened their ability to resist starvation

**Fig 1:**
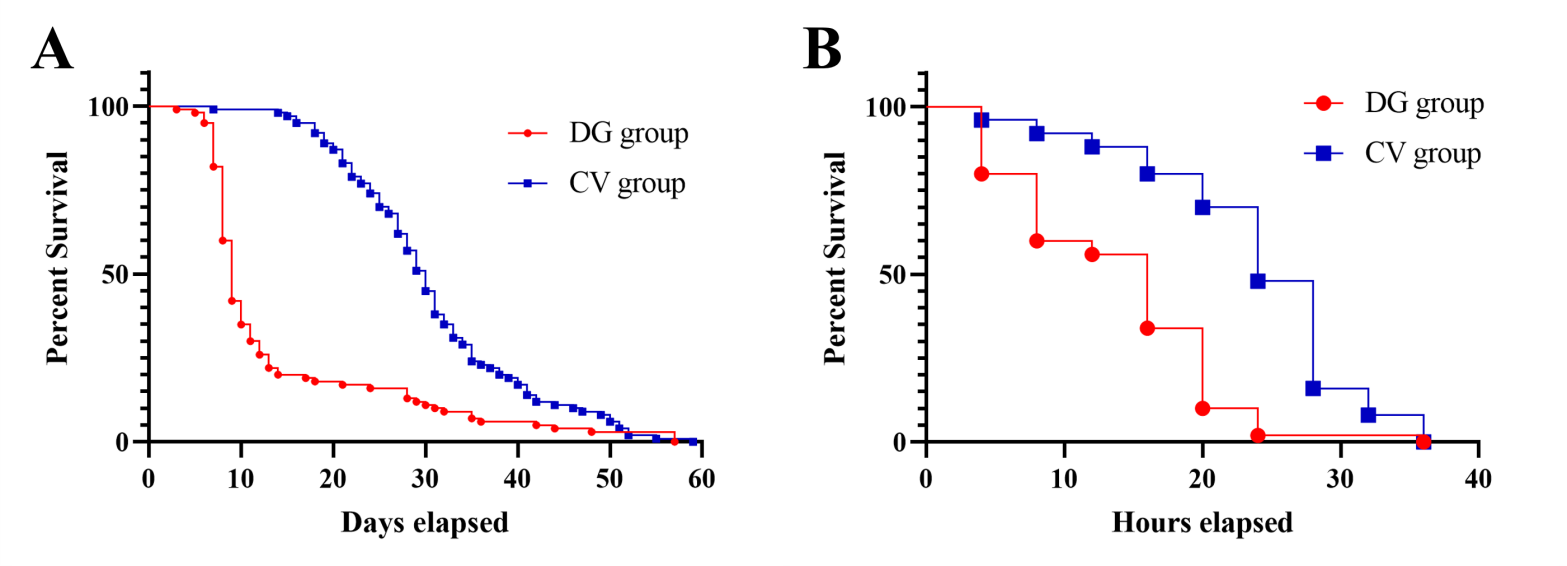
D-galactose treatment shortened the lifespan and impaired the energy storage ability of honeybees (A). The DG group was administered a D-galactose-supplemented sucrose solution (10% [wt/vol] D-galactose in 50% [wt/vol] sucrose solution). The CV group represents honeybees fed only sucrose (50%, wt/vol). Honeybees from the DG group have a shorter lifespan (*n* = 100; log-rank test, *P* < 0.001). (B) Honeybees from the DG group have a shorter lifespan in the starvation test (*n* = 100; log-rank test, *P* < 0.001).

Our investigation initially focused on evaluating the impact of D-galactose on honeybee longevity. Quantitative analysis revealed a significant reduction in mean lifespan following dietary supplementation with 10% D-galactose (*n* = 100; log-rank test, *P* < 0.001; Fig. 1A). Comparative survival analysis demonstrated substantial differences between CV and DG groups, with median lifespans of 30 (24–35) and 9 (8–13) days, respectively. To further characterize senescence-related phenotypes, we assessed energy homeostasis and stress resistance through controlled nutritional deprivation. On day 15 post-treatment, honeybees were subjected to sucrose and pollen deprivation while maintaining water access. This intervention resulted in complete mortality within 36 h, with the CV group exhibiting a median survival time of 24 (20–28) h compared to 16 (8–20) h in the DG group (*n* = 100; log-rank test, *P* < 0.001; Fig. 1B). These findings collectively demonstrate that D-galactose administration accelerates senescence in honeybees, manifesting as reduced longevity, impaired energy storage capacity, and diminished resistance to nutritional stress.

### D-galactose impaired the motor, learning, and memory abilities of honeybees

**Fig 2:**
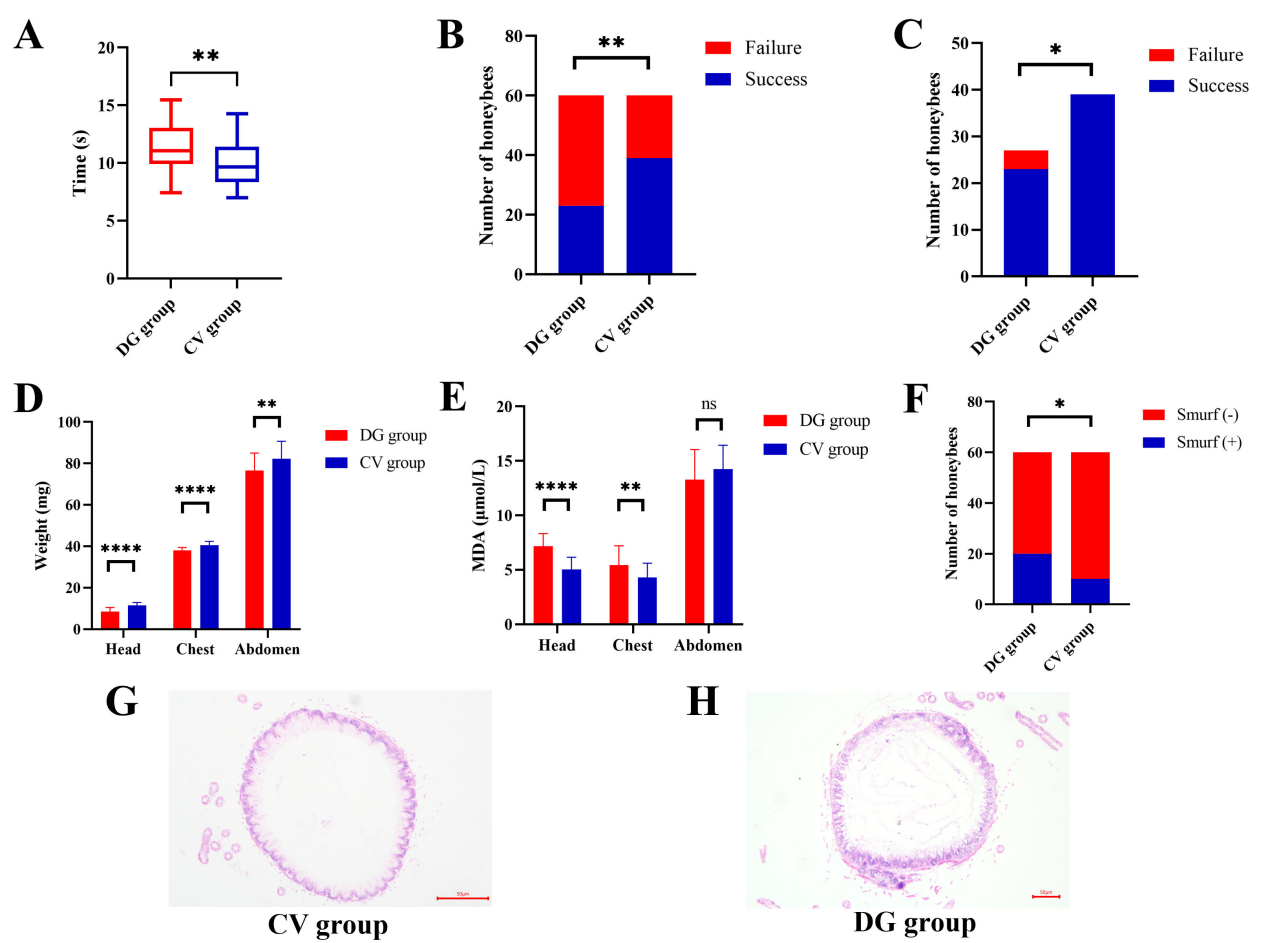
D-galactose treatment impaired the motor, learning, and memory abilities, decreased the weight, increased the MDA level, and disrupted the normal structure of honeybees. (A) The time to travel 50 cm is longer in the honeybees from the DG group (*n* = 50; *t*-test, **P < 0.01). (B) The number of honeybees who achieved success in the learning test is less in the DG group (*n* = 60; χ² test, **P < 0.01). (C) The number of honeybees who achieved success in the memory test is less in the DG group (*n* = 60; χ² test, *P < 0.05). (D) The weight of the head, chest, and abdomen of honeybees from the DG group is less (*n* = 50; *t*-test, ****P < 0.0001, **P < 0.01). (E) The MDA level of the head and chest is higher in the honeybees from the DG group (*n* = 50; *t*-test, ****P < 0.0001, **P < 0.01). The MDA level of the abdomen is similar in the two groups (ns, not significant). (F) The number of honeybees who achieved a positive result in the Smurf test is higher in the DG group (*n* = 60; χ² test, *P < 0.05). (G) Histopathologic evaluation with hematoxylin and eosin staining showed structural disruption in the epithelium of the midgut of the DG group.

Locomotor performance assessment revealed a significant impairment in DG honeybees compared to CV, with DG individuals requiring substantially more time to traverse a 50 cm linear track (11.36 ± 2.146 s vs. 9.988 ± 2.095 s; *n* = 50; *t*-test, *P* < 0.01; Fig. 2A). Cognitive function evaluation demonstrated marked differences in learning capacity, as evidenced by differential performance in conditioned stimulus recognition. Following five training trials, 65.0% of CV honeybees successfully acquired the conditioned response compared to only 45.0% in the DG group (*n* = 60; χ² test, *P* < 0.01; Fig. 2B). Memory retention testing further revealed compromised cognitive function in DG honeybees, showing significantly lower accuracy rates (85.19% vs. 100%; *n* = 60; χ² test, *P* < 0.05; Fig. 2C). These findings collectively demonstrate that D-galactose administration induces multifaceted behavioral deficits in honeybees, characterized by impaired motor function, diminished learning capacity, and reduced memory retention.

### D-galactose reduced the weight and increased the oxidative stress in honeybees

The heads, chests, and abdomens of 50 honeybees were individually weighed, as illustrated in Fig. 2D. CV honeybees exhibited significantly greater mass in all body segments compared to DG individuals: cephalic (11.49 ± 1.445 mg vs. 8.51 ± 1.915 mg; *n* = 50; *t*-test, *P* < 0.0001), thoracic (40.53 ± 1.837 mg vs. 37.96 ± 1.476 mg; *n* = 50; *t*-test, *P* < 0.0001), and abdominal (82.31 ± 8.380 mg vs. 76.66 ± 8.372 mg; *n* = 50; *t*-test, *P* < 0.01). To quantify oxidative stress levels, we measured MDA concentrations, a well-established biomarker of lipid peroxidation, in each body segment. The DG group demonstrated significantly elevated MDA levels in cephalic (7.161 ± 1.161 nmol/L vs. 5.043 ± 1.123 nmol/L; *n* = 50; *t*-test, *P* < 0.0001) and thoracic tissues (5.424 ± 1.786 nmol/L vs. 4.316 ± 1.299 nmol/L; *n* = 50; *t*-test, *P* < 0.01) compared to controls. However, no significant difference was observed in abdominal MDA concentrations between groups (14.24 ± 2.200 nmol/L vs. 13.28 ± 2.771 nmol/L; *n* = 50; *t*-test, *P* = 0.071; Fig. 2E). These findings demonstrate tissue-specific responses to D-galactose-induced oxidative stress, with particularly pronounced effects in cephalic and thoracic regions.

### D-galactose impaired the gut barrier of honeybees

The Smurf assay revealed a significantly higher prevalence of blue abdominal coloration in D-galactose-treated honeybees compared to controls (33.33% vs. 16.67%; *n* = 60; χ² test, *P* < 0.05; Fig. 2F), suggesting a compromised gut barrier function. Histopathological analysis of midgut tissue sections through hematoxylin–eosin staining demonstrated structural alterations in DG honeybees (Fig. 2G and H). Specifically, the DG group exhibited less mucin, thinner epithelium, and loss of epithelial integrity indicating a disruption of the normal structure.

### D-galactose altered the gut microbiome in honeybees

**Fig 3:**
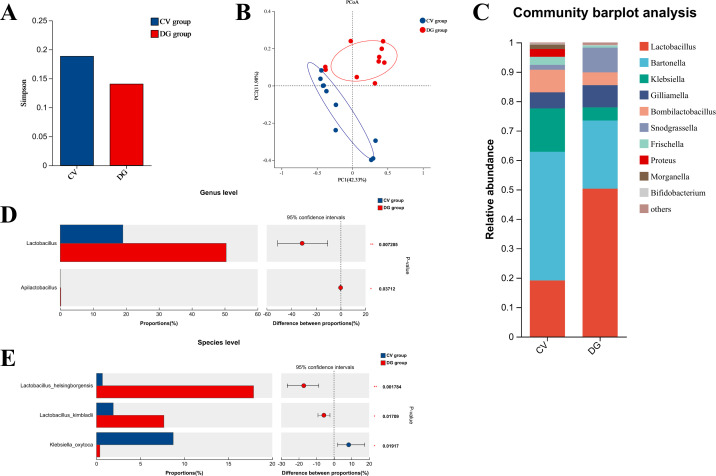
D-galactose treatment altered the gut microbiota of honeybees (A). The α-diversity is similar in the gut microbiota of two groups (*n* = 10, *P* = 0.345). (B) The β-diversity is different in the gut microbiota of two groups (*n* = 10, *P* < 0.01). (C) Gut microbiota composition of the honeybees in the two groups. (D) In the genus level, honeybees from the DG group had a higher percentage of *Lactobacillus n* = 10 (*P* < 0.01). (E) In the species level, *Lactobacillus helsingborgensis* and *Lactobacillus Kimbladii* were enriched significantly in the honeybees of the DG group (*n* = 10, *P* < 0.01, *P* < 0.05), together with the reduction of *Klebsiella oxytoca* (*n* = 10, *P* < 0.05).

Full-length 16S rRNA sequencing, accompanied by comprehensive bioinformatic analyses, was conducted on the samples from both the CV and DG groups. The findings demonstrated that the α diversity levels of the intestinal microbes were comparable between the two groups (*n* = 10; *P* = 0.345; Fig. 3A). Subsequently, an in-depth analysis of β diversity was carried out, which revealed that the composition of the gut flora in the DG group was significantly distinct from that of the CV group (*n* = 10; *P* < 0.01; Fig. 3B). This observation strongly suggests a potential influence of D-galactose in shaping the composition of the gut microbiota.

In the CV group, the top five most abundant bacterial genera in the gut were identified as *Bartonella*, *Lactobacillus*, *Klebsiella*, *Bombilactobacillus*, and *Gilliamella*. In contrast, for the DG group, the corresponding top five bacterial genera were *Lactobacillus*, *Bartonella*, *Snodgrassella*, *Gilliamella*, and *Bombilactobacillus* (Fig. 3C). Intriguingly, honeybees in the DG group exhibited a higher proportion of *Lactobacillus* compared to those in the CV group, whereas the CV group showed a higher relative abundance of *Klebsiella* (Fig. 3D). At the species level, a more detailed examination revealed that *Lactobacillus helsingborgensis* and *Lactobacillus kimbladii* were significantly enriched in the honeybees of the DG group, while the level of *Klebsiella oxytoca* was notably decreased (Fig. 3E). These results provide valuable insights into the differential distribution of bacterial species in the gut microbiota of honeybees under different experimental conditions.

### D-galactose changed gut metabolites in honeybees

**Fig 4:**
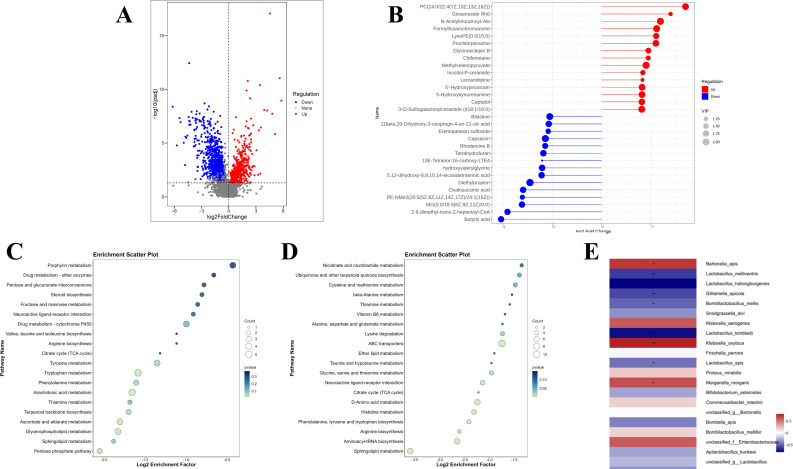
D-galactose treatment altered the gut metabolism of honeybees. (A) A total of 499 upregulated and 792 downregulated metabolites were identified in the gut of the honeybees from the DG group (*n* = 10). (B) Butyric acid was depleted in the DG group (*n* = 10, *P* < 0.05). (C) Upregulated metabolites of the DG group were concentrated in the pentose phosphate pathway, sphingolipid, and glycerophospholipid metabolism pathways. (D) Downregulated metabolites of the DG group were focused on the pathways of the sphingolipid metabolism, aminoacyl-tRNA, and amino acid synthesis, such as arginine, phenylalanine, tyrosine, and tryptophan. (E) The relative abundance of *Lactobacillus helsingborgensis* and *Lactobacillus Kimbladii* was significantly negatively correlated to the level of butyric acid (*n* = 10, ***P < 0.001), while there was a significant positive relation between butyric acid and *Klebsiella oxytoca* (*n* = 10, **P < 0.01).

The metabolic profiles of honeybees in the CV and DG groups exhibited substantial disparities. Through comprehensive analysis, a total of 499 upregulated metabolites and 792 downregulated metabolites were identified within the gut of honeybees from the DG group, as depicted in Fig. 4A. Among these differentially expressed metabolites, butyric acid was notably depleted in the DG group, as illustrated in Fig. 4B, suggesting a potential alteration in the gut metabolic environment. Furthermore, a Kyoto Encyclopedia of Genes and Genomes enrichment analysis was performed to explore the functional implications of these differential metabolites. When compared to the metabolite levels in the CV group, the upregulated metabolites in the DG group were predominantly concentrated in the pentose phosphate pathway, as well as in the sphingolipid and glycerophospholipid metabolism pathways (Fig. 4C). Conversely, the downregulated metabolites in the DG group were primarily associated with the sphingolipid metabolism pathway, along with the synthesis pathways of aminoacyl-tRNAs and various amino acids, including arginine, phenylalanine, tyrosine, and tryptophan, as presented in Fig. 4D.

Comparative analysis revealed significant alterations in both gut microbiota composition and intestinal metabolomic profiles in D-galactose-treated honeybees relative to controls. To elucidate potential interactions between microbial community structure and metabolic function, we performed Spearman correlation analysis of microbiota–metabolite relationships. This analysis identified significant negative correlations between butyric acid levels and the relative abundance of two *Lactobacillus* species, *L. helsingborgensis* and *L. kimbladii*, which were notably enriched in the DG group (*P* < 0.001). Conversely, a strong positive correlation was observed between butyric acid concentration and *Klebsiella oxytoca* abundance (*P* < 0.01), a species that was markedly depleted in DG honeybees (Fig. 4E). These findings suggest a complex interplay between specific microbial taxa and butyrate metabolism in D-galactose-induced senescence.

### Effects of D-galactose on honeybee genes related to learning, memory, immune response, and antioxidation

**Fig 5:**
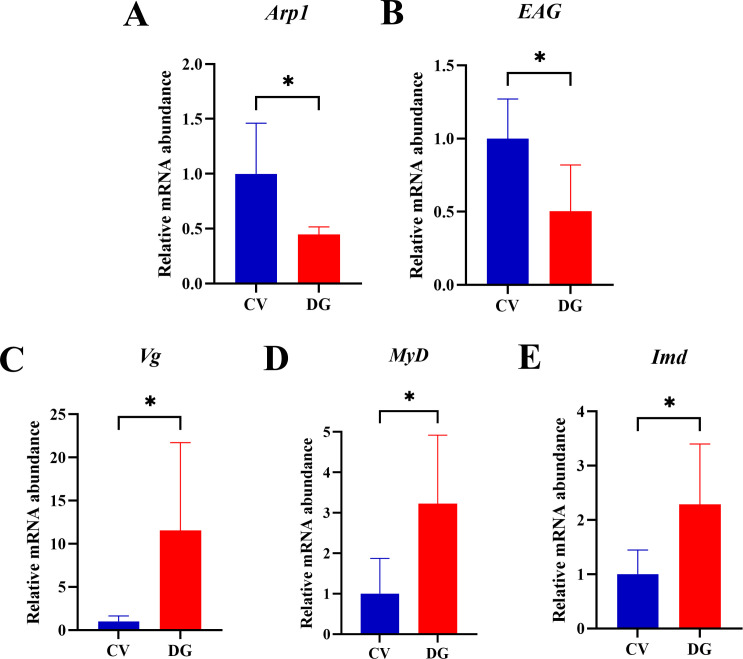
D-galactose treatment altered the gene expressions of honeybees. Intestinal tissues from the honeybees were homogenized for the RT-qPCR analysis. Differential mRNA levels of learning- and memory-related genes, (A) *Arp1* and (B) *EAG*; metabolic detoxification-related genes, (C) *Vg*; and immune system-related genes, (D) *MyD* and (E) *Imd*. Data shown are the results of five biological replicates per treatment (*n* = 5; *t*-test, *P < 0.05).

To elucidate the molecular mechanisms underlying aging-like behavioral regulation, we conducted quantitative analysis of transcript abundance for key honeybee genes associated with neurocognitive pathways (*actin-related protein 1* [*Arp1*] and *ether-à-go-go voltage-gated potassium channel* [*EAG*]) and stress response systems ([*itellogenin* [*Vg*], *immune deficiency* [*Imd*], and *myeloid differentiation primary response protein MyD88-A* [*MyD*]). Quantitative PCR analysis revealed distinct expression patterns between experimental groups (Fig. 5). The DG group exhibited a significant upregulation of *Vg* transcript levels compared to controls (*n* = 5; *t*-test, *P* < 0.05), accompanied by significant elevation in *Imd* and *MyD* (*n* = 5; *t*-test, *P* < 0.05) expression. Conversely, neurocognition-related genes demonstrated marked downregulation, with *Arp1* and *EAG* expression levels reduced significantly in DG specimens (*n* = 5; *t*-test, *P* < 0.05).

### Effects of butyric acid supplementation on honeybees treated with D-galactose

**Fig 6:**
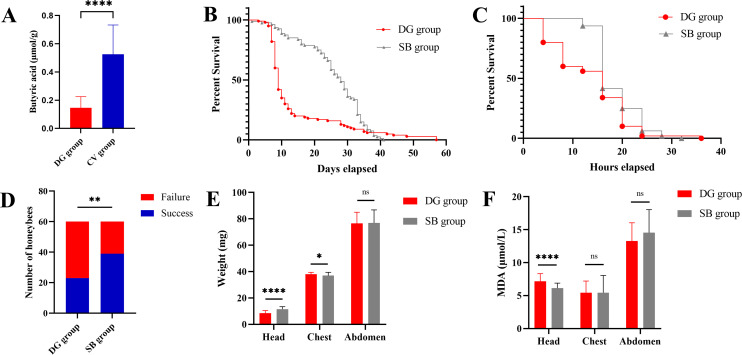
Supplementation of sodium butyrate ameliorated the impairment of D-galactose in honeybees. (A) The SB group represents honeybees fed sodium butyrate solution (sucrose solution [50%, wt/vol] supplemented with D-galactose [10%, wt/vol] containing 10 mM sodium butyrate). Honeybees in the DG group show a statistically significant reduction in the butyric acid concentration compared to the CV group (*n* = 10; *t*-test, *P* < 0.001). (B) Honeybees in the DG group have a shorter lifespan (*n* = 100; log-rank test, *P* < 0.001). (C) Honeybees in the DG group have a shorter lifespan in the starvation test (*n* = 100; log-rank test, *P* < 0.01). (D) The number of honeybees who achieved success in the learning test is less in the DG group (*n* = 60; χ² test, **P < 0.01). (E) The weight of the head of the honeybees from the SB group is heavier, and that of the chest is lighter (*n* = 50; *t*-test, ****P < 0.0001, **P < 0.01, ns, not significant). (F) The MDA level of the head is higher in the honeybees from the DG group (*n* = 50; *t*-test, ****P < 0.0001). The MDA levels of the head and abdomen are similar in the two groups (*n* = 50; *t*-test, ns, not significant).

To investigate the potential involvement of butyric acid in D-galactose-mediated aging processes in honeybees, we conducted quantitative analysis of butyric acid levels in both experimental groups. Our results revealed a statistically significant reduction (*n* = 10; *t*-test, *P* < 0.001; Fig. 6A) in butyric acid concentration in the DG group compared to the CV group, as determined by targeted metabolomic analysis. To establish a causal relationship between butyric acid depletion and observed senescence phenotypes, we implemented a sodium butyrate supplementation regimen in D-galactose-treated honeybees.

The SB group demonstrated significantly improved survival outcomes compared to the DG group. Kaplan–Meier analysis revealed a substantial extension in median lifespan, with SB-treated honeybees surviving 28 (21.5–33) days versus 9 (8-13) days in the DG group (*n* = 100; log-rank test, *P* < 0.0001; Fig. 6B). Under starvation conditions, SB-treated honeybees exhibited enhanced survival capacity, maintaining a median survival time of 16 (16–22) h compared to 16 (8–20) h in the DG group (*n* = 100; log-rank test, *P* < 0.01; Fig. 6C). Cognitive performance assessment demonstrated superior learning capacity in the SB group, with 65.0% successfully recognizing the conditioned stimulus compared to 45.0% in the DG group (*n* = 60; χ² test, *P* < 0.01; Fig. 6D). Biometric and biochemical analyses revealed tissue-specific responses to SB treatment (Fig. 6E and F). In cephalic tissues, SB-treated honeybees showed significantly higher mass (11.52 ± 1.854 mg vs. 11.49 ± 1.445 mg; *n* = 50; *t*-test, *P* < 0.0001) and reduced oxidative stress, as indicated by lower MDA levels (6.122 ± 0.776 µmol/L vs. 7.161 ± 1.161 µmol/L; *n* = 50; *t*-test, *P* < 0.0001). Thoracic measurements demonstrated reduced mass in SB-treated individuals (36.98 ± 2.523 mg vs. 37.96 ± 1.476 mg; *n* = 50; *t*-test, *P* < 0.05) with comparable MDA levels (5.444 ± 2.617 µmol/L vs. 5.424 ± 1.786 µmol/L; *n* = 50; *t*-test, *P* = 0.968). Abdominal analyses showed no significant differences in either mass (76.84 ± 9.968 mg vs. 76.66 ± 8.372 mg; *n* = 50; *t*-test, *P* = 0.926) or MDA levels (14.55 ± 3.491 µmol/L vs. 13.28 ± 2.771 µmol/L; *n* = 50; *t*-test, *P* = 0.072) between groups.

## DISCUSSION

Aging represents a complex biological process characterized by the progressive deterioration of physiological functions and cumulative cellular and tissue damage. Epidemiological studies have established significant correlations between human longevity and various sociodemographic factors, including marital status, social network complexity, and exposure to social stressors, underscoring the critical influence of social connectivity on aging trajectories (16–19). Concurrently, emerging evidence highlights the gut microbiome as a key modulator of aging-related physiological changes (20). The honeybee presents a unique model system for aging research, exhibiting sophisticated social organization manifested through resource allocation, caste-specific division of labor, and complex communication networks (21, 22). Notably, this eusocial species demonstrates remarkable lifespan plasticity, with reproductive queens surviving over 1 year, while non-reproductive workers typically live less than 1 month. This striking longevity disparity among genetically similar individuals within the same colony provides a distinctive paradigm for investigating the molecular and social determinants of aging (23, 24). In this study, we administered D-galactose to honeybees, a compound widely utilized in rodent models to induce accelerated aging for senescence-related studies. The D-galactose-treated honeybees exhibited characteristic senescence phenotypes, including significantly reduced lifespan, decreased body mass, diminished energy reserves, impaired cognitive function and locomotor capacity, dysregulated oxidative stress homeostasis, and compromised intestinal barrier integrity. Through comprehensive analysis of the gut microbiome, we identified a potential correlation between butyrate metabolism and the aging process, highlighting a crucial microbial–metabolic axis in senescence regulation.

Analysis of gut microbiota diversity revealed that there were significant differences in the gut microbiota composition between the CV and DG groups. Taxonomic profiling demonstrated a distinct microbial shift, with *Lactobacillus* emerging as the dominant genus in the DG group, contrasting with *Bartonella* predominance in the CV group. Notably, species-level analysis identified a significant enrichment of *Lactobacillus helsingborgensis* and *Lactobacillus kimbladii* in the DG group. This observation aligns with established literature demonstrating the antioxidative and stress-resistance properties of *Lactobacillus* species. Supporting evidence from Wang et al. (25) revealed similar *Lactobacillus* proliferation in honeybee gut microbiota following polystyrene microplastic exposure. Furthermore, experimental studies have documented that colonization with *Lactobacillus kunkeei* enhances detoxification capacity against deltamethrin in honeybees, suggesting a potential protective role of *Lactobacillus* species against environmental stressors. Extensive research has demonstrated that specific microbial strains can effectively mitigate multiple D-galactose-induced pathological manifestations, including oxidative stress, inflammatory responses, deterioration of motor and cognitive functions, and intestinal barrier dysfunction. The underlying mechanisms appear to be mediated, at least partially, through gut microbiome modulation, with butyric acid playing a pivotal regulatory role (26–29). Our findings suggest that D-galactose administration triggered a microbial community shift in honeybee gut microbiota, potentially as an adaptive response to counteract excessive oxidative stress. This ecological restructuring favored the proliferation of taxa with D-galactose metabolic capacity and antioxidative properties, particularly *Lactobacillus helsingborgensis*, a species with limited butyrate-producing capability (30, 31). Concurrently, the increased consumption of butyric acid, a crucial mediator of gut homeostasis and stress resistance, likely served as a compensatory mechanism against elevated oxidative stress. These dual processes—reduced butyrate production and increased metabolic utilization—collectively contributed to the significant depletion of butyric acid levels observed in the DG group, which may be intrinsically linked to the manifestation of senescence phenotypes.

Comparative analysis revealed that D-galactose-treated honeybees exhibited significantly reduced lifespan compared to sucrose-fed controls. Notably, this reduction was effectively ameliorated through sodium butyrate supplementation, suggesting a critical role of butyrate in counteracting D-galactose-mediated senescence processes. The lifespan-extending properties of butyrate are evolutionarily conserved, as evidenced by its ability to prolong longevity in *C. elegans* and *D. melanogaster*, and to recapitulate the beneficial phenotype observed in young germ-free mice. Potential mechanisms include activation of the DAF-16/FOXO signaling pathway and upregulation of heat shock proteins and fibroblast growth factor 21 expression (32–34). Furthermore, D-galactose-treated honeybees demonstrated reduced survival under starvation conditions, likely resulting from impaired energy homeostasis—a hallmark of normal aging processes. This metabolic dysregulation may be exacerbated by butyric acid deficiency. Supporting evidence from porcine models demonstrates that butyrate supplementation in gestating sows and piglets enhances weight gain and growth performance through modulation of lipid metabolism. Mechanistically, butyrate appears to suppress basal lipolysis while stimulating fatty acid oxidation, as indicated by increased expression of peroxisome proliferator-activated receptor gamma coactivator 1-alpha and peroxisome proliferator-activated receptor alpha in adipose tissue (35). This metabolic reprogramming promotes nutrient partitioning and enhances energy metabolism, ultimately leading to improved weight gain. Additionally, butyrate has been shown to enhance energy expenditure and partially restore microbiota depletion-induced thermogenesis impairment. These effects are potentially mediated through activation of the gut–brain axis and upregulation of uncoupling protein 1 expression, suggesting a systemic role of butyrate in energy homeostasis regulation (36).

A progressive decline in motor function represents a hallmark characteristic of aging organisms. Preclinical studies utilizing rodent models have demonstrated that D-galactose administration significantly impairs physical performance, as evidenced by reduced running duration, velocity, work capacity, and power output in treadmill exhaustion tests, coupled with diminished spontaneous locomotor activity (37, 38). Our findings are consistent with this established paradigm, showing that chronic D-galactose exposure similarly compromises motor function in honeybees, manifesting as reduced movement velocity and impaired locomotor capacity. While the precise mechanisms underlying this motor dysfunction require further elucidation, current evidence suggests that mitochondrial impairment and subsequent energy deficit in skeletal muscle tissue may play a pivotal role (39). The accumulation of reactive oxygen species leads to mitochondrial membrane potential depolarization, ultimately resulting in mitochondrial dysfunction. Notably, butyrate administration has been shown to mitigate this process through activation of the AMP-activated protein kinase-mediated mitophagy pathway (40). Furthermore, butyrate demonstrates therapeutic potential in ameliorating motor deficits associated with age-related pathologies, which could be attributed to multiple mechanisms, including the regulation of oxidation homeostasis and energy metabolism, as well as the enhancement of gut barrier integrity via the G-protein-coupled receptor 109A-nuclear factor kappa B pathway (41). This multifaceted action of butyrate underscores its potential in the treatment for age-related motor function decline.

Chronic exposure to D-galactose induced significant cognitive impairment in honeybees, recapitulating the learning and memory deficits characteristic of aging organisms (42, 43). Our experimental results demonstrated that D-galactose-treated honeybees exhibited impaired acquisition and retention of conditioned stimuli, accompanied by downregulation of cognition-related genes, indicating substantial deterioration of cognitive functions. This phenomenon is also evidenced by parallel findings in murine models, where D-galactose administration similarly compromised learning and memory capacities (44, 45). Notably, sodium butyrate supplementation partially attenuated these cognitive deficits, a finding consistent across multiple experimental systems (46, 47), underscoring the critical neuroprotective role of butyrate in cognitive processes. The beneficial effects of butyrate appear to be mediated through multiple molecular mechanisms (46, 47): (i) reduction of DNA damage frequency and enhancement of DNA repair capacity through upregulation of DNA repair genes; (ii) mitigation of neuroinflammatory responses; (iii) elevation of acetyl-CoA levels facilitating histone acetylation; and (iv) restoration of histone H3 lysine 9 acetylation and subsequent normalization of the brain-derived neurotrophic factor expression. These molecular alterations collectively contribute to the amelioration of butyrate on age-related cognitive decline, highlighting its potential as a therapeutic intervention for neurodegenerative conditions.

Extensive research has built a strong relationship between the D-galactose-induced senescence and oxidative stress pathways (48). In the present study, we quantified oxidative stress levels by measuring MDA, a well-established biomarker of lipid peroxidation. Our results revealed a significant increase in MDA levels in D-galactose-treated honeybees, with the exception of abdominal tissues. This tissue-specific response may be attributed to D-galactose-induced alterations in the gut microenvironment, where antioxidative metabolites, particularly butyric acid, were potentially mobilized to mitigate oxidative damage. Concurrently, we observed upregulation of genes associated with immune response and oxidative stress resistance, suggesting a compensatory protective mechanism. This finding aligns with previous reports highlighting the dual role of vitellogenin in honeybee immunity and antioxidant defense, particularly its capacity to protect genomic DNA against ROS-mediated damage (49, 50). Furthermore, when facing external stresses, such as pathogen infection or pesticide exposure, the expression levels of *MyD* and *Imd* were upregulated, which was also correlated with the level of MDA, as confirmed in other research (24, 51, 52).

Our investigation revealed an interesting finding that D-galactose administration compromised intestinal barrier integrity in honeybees, resulting in increased gut permeability and enteritis-like symptoms, which potentially contribute to reduced longevity. The underlying mechanisms appear to be multifactorial, involving both direct oxidative damage to cellular structures and barrier components, as well as indirect effects mediated through depletion of crucial regulatory molecules, such as butyrate (53, 54). Primarily, butyrate serves as the preferred energy substrate for colonocytes, providing energy requirements through β-oxidation, thereby supporting epithelial cell proliferation and differentiation (53). Furthermore, butyrate exerts regulatory effects on the mucus layer by stimulating goblet cells to increase mucin glycoprotein production (54). This results in enhanced mucus layer thickness and improved barrier function, collectively contributing to the prevention of bacterial translocation and regulation of intestinal permeability.

Butyric acid, a principal short-chain fatty acid (SCFA) derived from microbial fermentation of dietary fiber, has been widely recognized for its multifaceted beneficial effects on host physiology. In our experimental model, butyrate supplementation significantly attenuated D-galactose-induced senescence in honeybees, as evidenced by extended lifespan, restored energy homeostasis, and improved cognitive performance compared to D-galactose-treated controls. The immunomodulatory role of butyric acid in maintaining immune homeostasis has been extensively documented. Mechanistically, butyrate has been demonstrated to activate Toll-like receptor 2 (TLR2)-mediated TGF-β expression in colonic dendritic cells, thereby promoting regulatory T cell (Treg) differentiation and conferring protection against colitis (55). Furthermore, butyrate treatment significantly reduces the population of dendritic cells expressing T cell immunoglobulin and mucin domain 3(TIM3), a marker positively correlated with pro-inflammatory cytokine secretion (56). Alternatively, butyrate may directly facilitate the differentiation of Tregs via histone deacetylase inhibition, independent of dendritic cell-mediated TGF-β modulation (57). Another critical immunoregulatory mechanism involves the TLR2-dependent upregulation of interleukin-10, which further contributes to the anti-inflammatory effects of butyrate (58). The intricate interplay between gut microbiota and butyrate warrants significant scientific attention. Comparative analyses have revealed that Chinese long-living individuals exhibit a distinctive gut microbial profile characterized by an increased abundance of SCFA-producing bacteria and an enrichment of genes associated with butyric acid biosynthesis, in contrast to both elderly and younger populations (59). This observation is particularly relevant given that age-related declines in butyrate-producing bacteria have been shown to result in diminished butyrate levels and impaired free fatty acid receptor 2/3 (FFAR2/3) signaling. Such alterations can lead to compromised mucin production, increased intestinal permeability, systemic inflammation, and neurological abnormalities (60). Conversely, microbiome profiling through the BugBase database has demonstrated that methicillin-resistant *Staphylococcus aureus*-treated mice exhibit a marked increase in potentially pathogenic microorganisms, particularly within the *Erysipelotrichaceae*; however, butyrate pretreatment has been shown to effectively mitigate these microbial alterations (61). Furthermore, butyrate administration has been found to ameliorate rotenone-induced gut microbiota dysbiosis specifically by normalizing the *Firmicutes*/*Bacteroidetes* ratio—a microbial signature frequently associated with inflammatory conditions and implicated in the pathogenesis of Parkinson’s disease (62). While the precise molecular mechanisms underlying butyrate’s effects on senescence remain to be fully elucidated, current research strongly supports its pivotal role in modulating age-related physiological decline.

While this investigation provides novel insights into the interplay between gut microbiota and aging processes, several limitations should be acknowledged. Although honeybees share significant genetic conservation with humans in key metabolic and aging-related pathways, the considerable evolutionary divergence between insect and mammalian systems may limit direct translational applications. Besides, the current findings underscore the necessity for more comprehensive investigations into the complex interplay between gut microbiota, butyric acid metabolism, and aging processes.

In conclusion, this study demonstrates for the first time that honeybees exposed to D-galactose manifested senescence-like behaviors, providing a novel aging model. It reveals the effects of D-galactose on the enrichment of *Lactobacillus* and decrease of butyrate. Supplementation with butyrate partly reversed the effect of D-galactose, suggesting a critical mechanism underlying butyrate with senescence. In the future, honeybees could be further used for research into aging mechanisms and management through the regulation of gut microbiota, metabolites, or other active substances.

## Data Availability

The data sets generated during and/or analyzed during the current study are available from the corresponding author on reasonable request.
